# Multiplex T-RFLP Allows for Increased Target Number and Specificity: Detection of *Salmonella enterica* and Six Species of *Listeria* in a Single Test

**DOI:** 10.1371/journal.pone.0043672

**Published:** 2012-08-24

**Authors:** Geoffrey N. Elliott, Nadine Thomas, Marion MacRae, Colin D. Campbell, Iain D. Ogden, Brajesh K. Singh

**Affiliations:** 1 James Hutton Institute, Craigiebuckler, Aberdeen, United Kingdom; 2 Department of Medical Microbiology, University of Aberdeen, Foresterhill, Aberdeen, United Kingdom; 3 Hawkesbury Institute for the Environment, University of Western Sydney, Penrith, Australia; 4 Department of Soil and Environment, Swedish University of Agricultural Sciences, Uppsala, Sweden; Indian Institute of Science, India

## Abstract

A multiplex T-RFLP test was developed to detect and identify *Salmonella enterica* and all six species of *Listeria* inoculated into milk at minimal levels. Extensive *in silico* analysis was used to design a fifteen-primer, six-amplimer methodology and *in vitro* application showed target organism DNA, when amplified individually, yielded the predicted terminal restriction fragments (TRFs) following digestion. Non-target organisms were either not-amplified or yielded TRFs which did not interfere with target identification. Multiple target DNA analysis gave over 86% detection of total TRFs predicted, and this was improved to over 90% detection of total TRFs predicted when only two target DNA extracts were combined analysed. Co-inoculation of milk with five strains each of the target species of *S. enterica* and *L. monocytogenes*, along with five strains of the non-target species *E. coli* was followed by enrichment in SEL medium for M-TRFLP analysis. This allowed for detection of both target species in all samples, with detection of one *S. enterica* and two *Listeria* TRFs in all cases, and detection of a second *S. enterica* TRF in 91% of cases. This was from an initial inoculum of <5 cfu per 25 ml milk with a background of competing *E. coli* present, and gave a result from sampling of under 20 hours. The ability to increase target species number without loss of sensitivity means that extensive screening can be performed at reduced cost due to a reduction in the number of tests required.

## Introduction

The efficient and sensitive identification and detection of pathogen species in raw and processed foods as well as in other environments is fundamentally important to protecting human health. The days and sometimes weeks that a culture-based approach can take for the production of a trustworthy result has led to molecular approaches being investigated as being potentially more reliable, rapid and cost-effective. Continued use of the SSUrRNA gene in T-RFLP studies, specifically the 16s rRNA gene of bacteria, has allowed for the tentative identification of bacterial pathogens in many such cases, but the lack of diversity within this gene to the level of species, or a lack of consistent differentiation between species within genera, has limited its utility [1], [2], [3], [4]. Much research using genetic techniques such as monoplex, multiplex and quantitative PCR to test for the usefulness of a range of genes in the reliable identification of pathogen species has been performed to date [5], [6], [7], [8], [9], [10], [11], but the inability for multiple simultaneous target detection and resulting cost issues due to the need for individual tests to be performed for each pathogen has meant few approaches are economically viable e.g. in a four-dye qPCR system, only three targets are possible. Additionally, differences of much greater than a single nucleotide polymorphism between target sequences are required for specific probe binding, meaning that closely related species cannot in many cases be differentiated. Consequently, a novel test which allowed for a greater number of targets while retaining or enhancing specificity and reliability would be of value.

Terminal-Restriction Fragment Length Polymorphism (T-RFLP) analysis was first introduced to the scientific community as a methodology to identify species within a complex mix of bacteria and subsequently to identify particular isolates using the SSUrRNA gene [12], [13]. This methodology has since been primarily used in the analysis of the genetic diversity, change and composition of complex microbial communities within environmental samples [9], [14], [15]. There remained a continued focus on the SSUrRNA gene in these studies, its ubiquity allowing for a wide range of organisms to be amplified and analysed [16], coupled with a large available database allowing for subgroup targeting [17] and the easier interpretation of resulting data [18]. Since then, other ribosomal, housekeeping and functional sequences have been increasingly utilised, widening the scope of T-RFLP analysis into the diversity and change of various functional communities, including those of rhizosphere fungi and bacteria [19], [20], marine denitrifiers [21], ammonia-oxidisers [22], methanogens [23], methanotrophs [24] and plant pathogen-suppressing pseudomonads [25] amongst several others. T-RFLP analysis has also been applied to community analysis in industrial processes [22], [26] and in medical studies [22], [27], [28]. Multiplex T-RFLP (M-TRFLP) was first introduced in 2006 [29] as a novel culture-independent method to study multiple biomarkers of microbial communities simultaneously, and was subsequently suggested [30] as a potential tool in microbial diagnostics, allowing for wide ranging detection of a varied number of target sequences to indicate the presence and/or the functional capability of the target organisms. Studies have utilised this approach in its environmental context [31], but not in the area of diagnostics.

The goal of this study was to develop a novel detection technology based on M-TRFLP [32], to detect and identify multiple pathogens in a single test targeting multiple genes in order to provide a rapid, reliable and cost-effective tool. For this study we targeted *Salmonella enterica* and *Listeria* spp., commonly tested for in the food-production environment (ca. 129 million tests worldwide in 2008 [33]) and measured down to the lowest regulated standards (absence in 25 g [34]), and highlight a general approach for use in other applications for the identification of particular species in complex samples.

## Results

**Figure 1:**
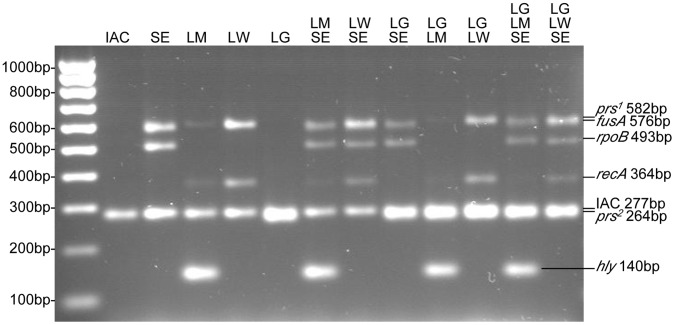
Multiplex Polymerase Chain Reaction amplification products shown following gel electrophoresis. Lanes 1 and 12 contain HyperLadder V (Bioline), with the remaining lanes containing products derived from 6 ng genomic DNA from as shown. Internal amplification control (IAC) template DNA was included in all reactions. Templates were amplified in isolation (apart from universal inclusion of the IAC) or in combination as described in the figure using a 15-primer multiplex PCR. Codes used are as follows: SE, *Salmonella enterica* MISE807439; LM, *Listeria monocytogenes* CMCC2993; LW, *Listeria welshimeri* CMCC3366; LG, *Listeria grayi* CMCC3362. Patterns for *L. seeligeri*, *L. murrayi*, *L. ivanovii* and *L. innocua* were identical to that of *L. welshimeri*, and all patterns shown were representative of all other strains tested of the same species. Size standards are descirbed on the left of the figure in base pairs (bp), while PCR products are described on the right of the figure with both their name and size in bp. ^2^ indicates the *prs* amplimer from *Listeria grayi* only, with ^1^ denoting the corresponding product from all other *Listeria* species. Electrophoresis was performed on a 1.7% agarose gel at 70 volts for 1.5 hrs with EtBr, with 4 ul of each product loaded.

For the identification of *Salmonella enterica* (SE), both *fusA* and *rpoB* gene sequences were selected and primers designed (Table 1) amplified the correct fragment size in the presence of SE DNA (Figure 1). In both cases we were unable to design oligonucleotide primers that were certain of not annealing to the sequences of related Enterobacteriaceae e.g. *Escherichia coli* (EC), *Shigella* spp., *Enterobacter* spp. without impeding the amplification of some target SE. However, primers were designed to ensure all SE were included rather than exclude some SE along with these non-target organisms, instead using fragment sizing to exclude these. Of the non-target species tested, the *rpoB* and the *fusA* primers gave PCR amplification products only with the genera *Citrobacter*, *Enterobacter*, *Klebsiella*, *Escherichia*, *Proteus*, *Yersinia* and *Shigella* for one or both sets of primers. However, no TRF produced from any non-target strain amplified using either primer set were of sizes that matched the corresponding SE TRFs produced.

**Table 1:** Genes targeted and oligonucleotide primers designed.

Gene	Target genera/species	Name	5′-3′ oligonucleotide primer sequence	A	B	Size (bp)
*fusA*	*Salmonella enterica*	FusSalFwd	CCA TCA TCG CTG GTA TGG G	−	−	576
		FusSalRev	CAG ATT CCT GAC CTT TGA GC	HEX	FAM	
*hly*	*Listeria monocytogenes*	HlyFwd2	GCC TGC AAG TCC TAA GAC G	FAM	FAM	140
		HlyRev2	AAC CTT TTC TTG GCG GCA CA	FAM	FAM	
pGEM-	Internal amplification control	IacFwd	ACG ACT CAC TAT AGG GCG	ROX	ROX	277
3Zf (+)	(IAC)	IacRev2	ACG ACA GGT TTC CCG AC	None	None	
*prs*	*L. innocua; L. ivanovii;*	PrsListFwd*	GCC TTA CTA TGG **Y**TA **Y**GC ACG	TET	PET	582
	*L. monocytogenes; L. seeligeri;*	PrsListRevA*	TCA ATC CAT TT**K** TCT TCT GGA AGA GC	TET	NED	
	*L. welshimeri*	PrsListRevB	TCA ATC CAT TTA TCT TCT GGG AGA GC	TET	NED	
*prs*	*L. grayi*	PrsListFwd2	ACC GCG TCC GAA TGT CGC A	TET	PET	264
		PrsListRevC	TCG ATC CAT TTT TCT TCT GGT AAA GC	TET	NED	
*recA*	*L. innocua; L. ivanovii;*	RecListFwd3*	CC**W** GAT ACA GGA GA**R** CAA GC	FAM	FAM	364
	*L. monocytogenes; L. seeligeri;*	RecListRev*	TAC CCA TTA CAT C**H**G TAC CTT G	FAM	FAM	
	*L. welshimeri*					
*rpoB*	*S. enterica*	RpoSalmFwd2	GCG TAC CTA ACG GTG TC	ROX	VIC	493
		RpoSalmRevB	TCG ATC GGG TTG ATC TTA GAG ATA	ROX	VIC	

* Degenerate primers, where degeneracies follow the IUPAC nomenclature for incompletely specified bases and are described in bold, specifically: ‘Y’ = C or T; ‘K’ = G or T; ‘W’ = A or T; ‘R’ = A or G; ‘H’ = A, C or T. (www.chem.qmul.ac.uk/iubmb/misc/naseq.html).

# Primers were 5′ labelled with fluorescent dyes according to the text using either complete dye set A or B.

Target *Listeria* species in this analysis were namely *L. monocytogenes* (LM), *L. ivanovii* (LV), *L. innocua* (LI), *L. seeligeri* (LS), *L. welshimeri* (LW) and *L. grayi* (LG), with amplification of the *prs* gene used for differentiation of all six species of *Listeria*, the *recA* gene for further identification of all *Listeria* species apart from LG, and the *hly* gene for specific identification of LM (Figure 1). *In silico* analysis of the combination of gene sequences used to differentiate *Listeria* spp. indicated that all species should be discernible from one another, with unique TRFs present for all but one of the seven target species (Table 2).

**Table 2:** Terminal restriction fragments (TRFs) predicted and produced from five target genes following individual species amplification and *Hha*I digestion.

	*fusA*	*hly*	*recA*		*rpoB*		*prs*		TRF Profile	Unique (total)	
Species tested	DTRF	Uncut	UTRF	DTRF	UTRF	DTRF	UTRF	DTRF	(unique TRFs in bold)	TRFs	
**PREDICTED:**											
***S. enterica***	hex458*	–	–	–	rox226*	rox121*	–	–	hex**456**/rox**121,226**	3 (3)	
***L. monocytogenes***	**–**	fam140*	fam288*	fam78	**–**	**–**	tet292	tet132	fam78,**140,288**/tet132/292	2 (5)	
***L. innocua***	**–**	**–**	fam55	fam78	**–**	**–**	tet292	tet132	fam55,78/tet132/292	0 (4)	
***L. grayi***	**–**	**–**	**–**	**–**	**–**	**–**	tet134*	tet132	tet132/**134**	1 (2)	
***L. ivanovii***	**–**	**–**	fam55	fam78	**–**	**–**	tet127*	tet132	fam55,78/tet**127**/132	1 (4)	
***L. seeligeri***	**–**	**–**	fam55	fam263*	**–**	**–**	tet292	tet292	fam55,**263**/tet292	1 (4)	
***L. welshimeri***	**–**	**–**	fam103*	fam82*	**–**	**–**	tet265*	tet292	fam**82,103**/tet**265**/292	3 (4)	
**MEASURED:**											
***S. enterica***	hex453*	**–**	**–**	**–**	rox225*	rox117*	**–**	**–**	hex**453**/rox**117,225**	3 (3)	
***L. monocytogenes***	**–**	fam138*	fam286*	fam73^a^	**–**	**–**	tet291	tet129^b^	fam73,**138,286**/tet129/291	2 (5)	
***L. innocua***	**–**	**–**	fam51	fam73^a^	**–**	**–**	tet291	tet129^b^	fam51,73/tet129/291	0 (4)	
***L. grayi***	**–**	**–**	**–**	**–**	**–**	**–**	tet133^b^	tet129^b^	tet129/133	1 (2)	
***L. ivanovii***	**–**	**–**	fam51	fam73^a^	**–**	**–**	tet125^b^	tet129^b^	fam51,73/tet125/129	1 (4)	
***L. seeligeri***	**–**	**–**	fam51	fam262*	**–**	**–**	tet291	tet129^b^	fam51,**262**/tet129/291	1 (4)	
***L. welshimeri***	**–**	**–**	fam99*	fam75^a^	**–**	**–**	tet262*	tet288*	fam75,**99**/tet**262**/**288**	3 (4)	

All TRFs are listed using the code: (dye)(length in bp).

* TRFs unique to that species and definable on analysis.

a TRFs combined into a single bin due to lack of distinction on analysis.

b TRFs combined into a single bin due to lack of distinction on analysis.

UTRF: TRF derived from the upstream i.e. forward primer end of the amplicon.

DTRF: TRF derived from the downstream i.e. reverse primer end of the amplicon.

*In vitro* analysis using primers labelled with set A dyes (Table 1) showed that most of the different peak sizes (9 of 16) were sized within expected parameters of variation for size measurement on the DNA sequencer (2–3 bp difference) although some larger differences were observed. Only one fragment, the *prs* DTRF of LS, did not match the general peak size predicted. Rather, the peak detected was the same size as the corresponding fragment of the other four *Listeria* species.

**Figure 2:**
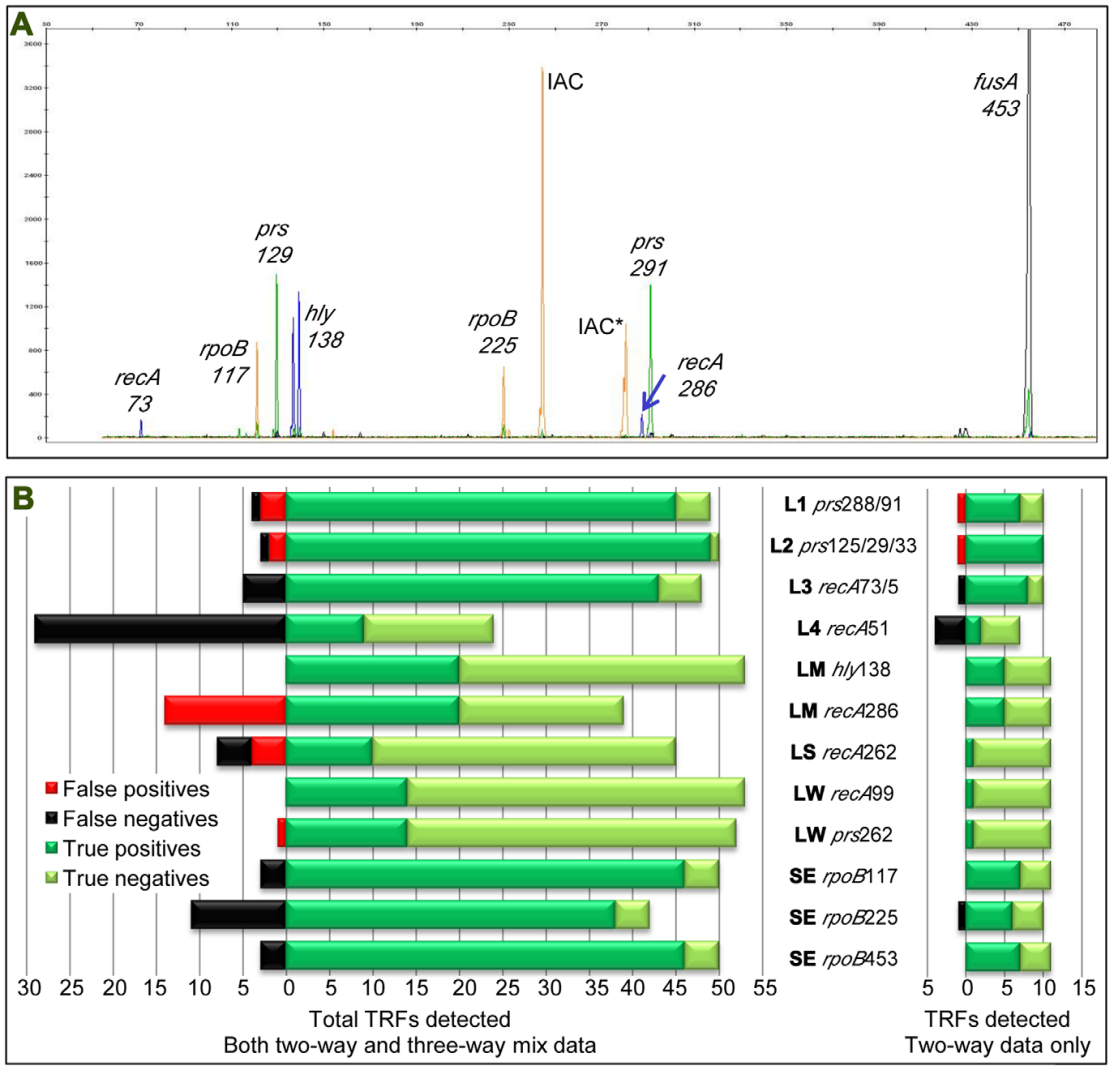
M-TRFLP profile and analysis of pre-amplification DNA template mixes. A: M-TRFLP profile of pre-enrichment mix of five strains of each of *L. monocytogenes, S. enterica* and *E. coli*, following Chelex extraction of DNA, multiplex PCR, post-PCR cleanup and *Hha*I restriction digestion. TRFs are labelled as the genes they represent and the sizes reported in base pairs, with *rpoB* and *fusA* TRFs *S. enterica* specific and TRFs from *recA*, *hly* and *prs L. monocytogenes* specific. Inocula were mixed in the ratio of 1∶1:10 in favour of *Listeria*, and DNA extracted from the sample post-enrichment using a Chelex-based procedure prior to PCR amplification and the products purified following PCR and prior to restriction digestion with *Hha*I. The TRF labelled IAC is the internal amplification control, and the IAC TRF with the asterisk undigested. B: TRF detection from two- and three-way pre-amplification template mix experiments. True and false positives and true and false negatives are indicated. Total specific TRF detection for all data is presented on the left and just two-way mix data on the right. The presence of bacteria specifically indicated by each TRF are coded as follows: SE, *S. enterica*; LG, *L. grayi*; LI, *L. innocua*; LV, *L. ivanovii*; LM, *L. monocytogenes*; LS, *L. seeligeri*; LW, *L. welshimeri*. *Indicates more than one target *Listeria* species is indicated by the presence of this TRF: L1 indicates the presence of LI, LM, LS, or LW; L2 indicates LG, LI, LV, LM, or LS; L3 indicates LI, LV, LM, or LW; L4 indicates LI, LV, or LS.

So as to assess detection from complex multi-target samples, a matrix of template mixes was then tested, consisting of multiple combined target templates (two-fold or three-fold combinations) from the one SE and six *Listeria* spp. at varying relative concentrations, along with a standardised concentration of an Internal Amplification Control (IAC) (Table 1; Figure 1). On M-TRFLP analysis, the *prs* gene TRFs previously measured at 125, 129 and 133 bp were difficult to distinguish in mixed samples and were therefore grouped into one individual class or ‘bin’, encompassing all three TRF sizes, for all further analysis. This impacted the ability of the test in its current form to differentiate LG and LI, as the ‘unique’ TRFs for both target species fell within this bracket. The TRFs 288 and 292 as well as the TRFs fam73 and 75 similarly were difficult to distinguish in mixed samples and so again were combined into group bins. However, with other unique markers for the detection of LW, this did not impact identification. It was also apparent that low or non-amplification of certain products in some mixed profiles had sporadically occurred (Figure 2), notably, the 51 bp *recA* TRF that was expected from those samples containing DNA from LI, LV and LS performed particularly badly, with only 23.7% of those samples found containing that TRF (9 of 38 samples).

The average percentage of TRFs detected compared to those expected across all of these combined samples was 86.1% (not including the IAC, which was amplified in all cases), with 354 of 411 expected peaks detected, and a further 25 spurious peaks found (i.e. peaks expected from other target species not present in the sample analysed) (Figure 2). These data as a whole showed that in terms of ‘unique’ peaks, LW was always detected and never gave a false positive (with respect to its ‘unique’ *recA* 99 TRF). The same species was similarly always detected using *prs* 262 but one sample (of 39) not containing LW gave a false positive. LM was always detected using the *hly* 138 TRF without any false results, and the *recA* 286 TRF peak similarly was detected 100% of the time, but gave 42% false positives from those samples without LM but with other *Listeria* species. The remaining TRFs were detected in less than 100% of all samples, although five of those eight remaining TRFs gave detection rates of over 89%. In one sample of the 65 tested was only one TRF detected of an expected eight (apart from the IAC), giving a 13% detection rate (Figure 2). The next lowest detection rate for an individual sample was 50% (3 of 6 peaks detected), and the overall mean peak detection rate was 85% (+/-16%), with 17 samples of the 53 showing all TRFs expected and no spurious TRFs (Figure 2).

On removal of the three-way template mix samples (those containing three target species) from the dataset, results were improved in terms of TRF detection (Figure 2). Although the *recA* 51 UTRF still performed badly (33.3% detection rate), the mean percentage of bands detected compared to those expected across all of these combined samples was 90% (s.d. +/-12%), with 60 of 66 expected peaks detected, and only 2 spurious peaks found (0.3% of total). SE, LM, LS and LW were specifically detected in 100% of these two-way mixes in terms of their unique TRFs, with no false positives, and three of these four had two unique markers independently confirming the result (Figure 2). The test again could no longer differentiate the remaining three *Listeria* spp. from other *Listeria* spp. due to the combination of several *prs* TRFs into one bin as stated previously - the presence of *Listeria* spp. was all that could be indicated. This said, either spurious peak detection (both *prs* TRFs indicating a small number of false positives) or a lack of total detection (less than 100% detection for the *recA* 73/75 TRF, the *recA* 51 TRF and the *rpoB* 225 TRF) reduced the utility of these TRFs in isolation.

### Food Matrix M-TRFLP Testing

The M-TRFLP approach was transferred to a live-cell-based methodology to determine if it could be similarly successful following co-enrichment of target organisms with each other and with non-target competing organisms at varying SE:EC:LM ratios. Inoculated milk was added to enrichment media to give a final (pre-growth) inoculated media colony forming unit count of 176 cfu/L, 129 cfu/L and 216 cfu/L for five strain mixes of SE, EC and LM respectively for the high inoculum concentration and ten-fold less than this for the low inoculum concentration. Following enrichment, both target species were detected in all cases using standard culture, and equated to 4.4, 3.2 and 5.4 cfu per 25 ml of milk respectively for the lowest level of initial inoculum.

**Figure 3:**
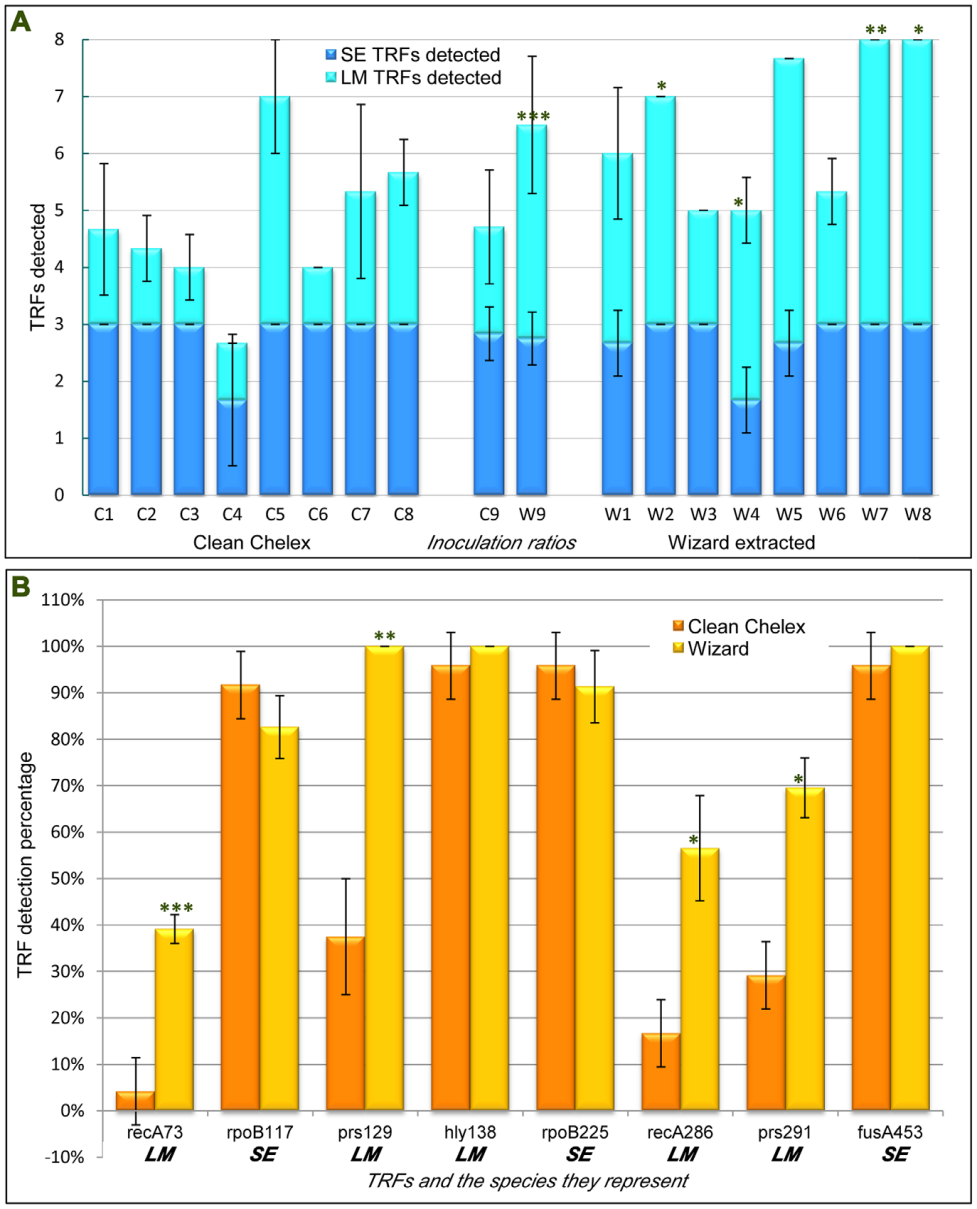
Terminal Restriction Fragment (TRF) detection for DNA extraction and post-amplification clean-up protocols for inoculated milk experiments. A: TRFs detected for both *S. enterica* (SE) and *L. monocytogenes* (LM), and are indicated as described in the legend, with three SE TRFs and five LM TRFs expected for all samples. TRFs detected are shown for the mean averages of three individual replicates in each case. Error bars shown are +/-1SD for both TRFs detected values. Milk was inoculated with five strains each of *S. enterica* (SE), *E. coli* (EC) and *L. monocytogenes* (LM) at 4.4, 3.2 and 5.4 cfu per 25 ml of milk respectively for the lowest level of initial inoculum (i.e. 10^−7^ dilutions) and tenfold this for 10^−6^ dilutions. These were used to make up inoculum ratios, shown as either Cn or Wn, where C indicates Chelex extracted and cleaned samples, W indicates Wizard extracted and uncleaned samples and n indicates ratios (SE:EC:LM respectively) as follows: 1, 1∶1:1 (10^−7^ dilutions); 2, 1∶1:1 (10^−6^ dilutions); 3, 10∶1:1; 4, 1∶10:1; 5, 1∶1:10; 6, 10∶10:1; 7, 10∶1:10; 8, 1∶10:10; 9, mean average of all corresponding samples. Paired student’s two-tailed t-tests were performed between all 9 corresponding C and W datasets for both SE and LM TRF types e.g. C1 LM TRF figures were paired and compared with W1 LM TRF figures. Asterisks at the top of the corresponding W bar indicate significant differences, with one asterisk indicating a P value of <0.02, two indicating P<0.01, and three indicating P<0.0001. B: Total specific TRF detection percentages across DNA extraction and post-amplification product clean-up treatments for pre-enrichment inoculum ratio experiments as above. Specified TRFs detected for both *S. enterica* (SE) and *L. monocytogenes* (LM), and are indicated as described. Error bars shown are +/-1SD for all data, derived from triplicate datasets. Paired student’s two-tailed t-tests were performed between corresponding Clean Chelex and Wizard extracted DNA datasets for all eight TRFs. Asterisks at the top of the corresponding bar indicate significant differences, with one asterisk indicating a P value of <0.05, two indicating P<0.02, and three indicating P<0.005.

Following Chelex extraction and post-PCR cleaning as standard, an average of 2.8 of a maximum of 3.0 TRFs were detected across all samples for SE but just 1.8 of a maximum of 5.0 TRFs detected for LM across all 24 replicates (Figure 3). All three expected SE TRFs were detected in 22 of 24 replicates, the remaining two replicates with just one SE TRF detected. All five LM TRFs were detected in just one replicate. Four out of five LM TRFs were detected in a further two replicates, four more had three LM TRFs, three had two LM TRFs, and thirteen replicates indicated the presence of just one LM TRF of the five expected (Figure 3). These results equated to an average of 94.4% peak detection for SE and 36.7% peak detection for LM. No single TRF was detected in all sample replicates. Three of the eight expected TRFs were detected in all but one replicate, and a further TRF was detected in all but two replicates, these four TRFs being the *hly* TRF of LM, and the *fusA* and both *rpoB* TRFs of SE. The remaining four TRFs all had detection levels of nine of 24 sample replicates or below, and all of these were LM indicators (Figure 3).

Alterations in the DNA extraction methodology, from a Chelex-based extraction to one based on Wizard, and removal of the post-PCR cleaning step were decided upon in an attempt to further boost detection levels. All samples were subsequently re-amplified. In one replicate the IAC could not be detected and so was discarded. Of the remaining 23 sample replicates, all three SE TRFs were detected in 18 replicates, with four of the remaining five replicates showing the presence of two SE TRFs and the last showing one only. All five LM TRFs were detected in nine replicates, with a further four replicates presenting four of five LM TRFs. Of the remaining 10 samples, three indicated the presence of three LM TRFs, and the remaining seven showed two LM TRFs. These results equated to an average of 91.3% peak detection for SE and a 73.0% peak detection for LM for this treatment. In terms of specific TRF detection, three TRFs were detected in all 23 samples, two indicating LM and the other SE, with a further SE TRF detected 91% of the time. The lowest detected TRF was the *recA* 73 TRFas before, with 39.1% detection. The remaining TRFs were all detected in at least 13 of 23 samples.

Although dye set A was selected due to these dyes being non-proprietary, an alternative dye set, ‘B’, containing proprietary dyes was tested. Using PCR cleaned samples for both Chelex and Wizard extracted DNA, all 3 SE TRFs were detected in 22 of the 24 Chelex extracted replicates (97%), and 21 of the 23 Wizard extracted replicates (95%) showed the same. However, only 23% and 33% of the corresponding LM TRFs were detected. Also, in both cases, two of 24 samples tested gave no IAC detection and so were discarded in the analysis. A further two samples from the Chelex-extracted set and three from the Wizard-extracted set gave no detection of any LM TRFs. Thus ABI dyes were not used for extended analysis (data not shown).

## Discussion

While this study follows work performed using T-RFLP on 16s rRNA genes to identify species in simple culture [2], [13], our simultaneous analysis of multiple sequences using M-TRFLP for specific detection is novel [32]. The ITS sequence has been used several times in combination with the 16s rRNA genes in the past [24], [29], [31] but always in community analysis rather than specific identification of species. Monoplex T-RFLP approaches identifying pathogenic species in isolation [2], [4], [35], [36] or as members of mixed communities [1], [12], [37] are published, but have either been limited by their monoplex nature or by the necessity for multiple monoplex amplifications and digestions to differentiate the organism in question to the point required. In many cases the analysis of a complex bacterial mix confounds the certain identification of species when particular combinations (i.e. profiles) of more common traits are required to identify target species, rather than individual and unique traits.

With *Salmonella* one of the most common causes of food-borne illness, and Listeriosis a serious illness for those with heightened susceptibility, ca. 69 million tests were performed for *S. enterica* and ca. 60 million *L.monocytogenes/Listera* spp. tests were performed worldwide in 2008 [33]. Our aim was to identify the presence of *S. enterica* along with the six different species of *Listeria* in one sample. This approach allows for co-culture to be used, as re-combination of samples following culture in isolation is impractical and costly. Although it is *L. monocytogenes* that is associated with serious foodborne illness, it is recognised that an effective way to prevent *L. monocytogenes* contamination is to monitor *Listeria* spp. in general at all stages of food production as an indicator of the conditions necessary to allow *L. monocytogenes* contamination to occur.

The success of our M-TRFLP approach on individual strain amplification led to the use of mixed templates in various ratios, allowing for the optimisation of primer concentration and other methodological factors. This allowed us to maximise the numbers of TRFs detected in samples approximating the complex target mixes that might be found in a ‘real-life’ sample. We worked with an aim of always being able to detect *S. enterica*, *L. monocytogenes* and at least one further *Listeria* sp., or at least two *Listeria* spp. (as well as *S. enterica*) in the absence of *L.monocytogenes* and down to the legislated minimum cell numbers across all foods i.e. the absence of both organisms in 25 g of product [34]. In real terms, the presence of just one pathogenic species would be sufficient to warrant further confirmatory testing, but our approach allowed for a higher stringency and an acceptable level of within-genera redundancy for *Listeria*. This said, both false negatives and false-positives were found in both two-way and three-way mixes. There was a reduction of these when the more complex three-way mixes were removed from the analysis, but not to the point of removal of these false results, and this may well have been partly due to the smaller numbers of samples within the two-way dataset. However, seven of the twelve TRFs gave perfect results within this dataset, and a result would be more assured if only this subset of TRFs were considered. False positives were derived from the amplicons of sister *Listeria* spp., supported by the lack of false positives for any *S. enterica* TRF. These may be due to incomplete digestion of amplicons rather than due to pseudo-TRF formation which can occur with the complex secondary structures common to 16s rRNA sequence analysis [38]. Such incomplete digestion would be more obvious in our approach than in community analysis, and thus may be more common than assumed. Alteration of digestion conditions may alleviate this problem. The presence of false TRF negatives were countered in all cases by the presence of significant numbers of true positives, allowing for the presence rather than the absence of TRFs to take precedence in the identification process.

Differences in TRF sizes predicted led to the need to group (i.e. “bin”) several different TRFs initially seen as unique and allowing for the differentiation of some *Listeria* spp. from others. Thus differentiation was reduced, and three of the seven species lost their unique identifier. In all cases these species would only not be able to be definitively identified in the presence of other sister species of *Listeria*, due to some common TRFs, so each species remained unique in terms of their entire TRF profiles, and these were found in isolation for all strains. Although no confirmatory sequencing was performed to investigate differences between predicted and reported TRF sizes, it was assumed that the sequences reported in *in silico* databases were correct. This said, large-scale differences between the *prs* fragment sizes of *L. seeligeri* sequences in the database and that of one of our *L. seeligeri* was assumed to be a genuine genetic variation. As *recA* sequences were specific and unique to *L. seeligeri* there was no question as to whether this was a strain of this species and this variation was seen as a genuine, within species, point mutation.

Fluorescence dyes used in multiplex analyses (i.e. dye set A) were selected on the basis of their non-proprietary nature i.e. these dyes were not patent-protected and therefore could be used in a commercial sense without further consideration. Use of some proprietary dyes (dye set B) did allow improved differentiation of binned TRFs, and removed an amount of peak ‘pull up’ that we found, but gave slightly worse results overall when used in milk experiments. This may well be due to the fact that amplification was optimised with set A dyes and it has been reported that the use of different dyes can have an effect on the PCR reaction [39]. It may be that use of dye set B and optimisation with these dyes could improve the process overall and allow for more distinct separation of the binned TRFs. However, the TRFs within these combined bins still performed relatively poorly in terms of detection levels.

Milk was selected as a liquid matrix for ease of achieving an even distribution of inocula, yet is a widely used and therefore microbiologically regulated food substance, utilised both in a relatively unadulterated state and as a precursor or ingredient to many other foods. This application does not preclude the use of this method with other sample types. In terms of inoculum concentrations used for the milk-based experiments, one cell in 25 ml can be expected to generate 40% true negatives, and so we aimed for 3 to 5 cfu/25 ml sample so as to reduce or eliminate the levels of true negatives encountered. The test levels of 4.4 and 5.4 cfu per 25 ml of milk for *S. enterica* and *L. monocytogenes* respectively filled this criteria and thus addressed the European commission regulations for a test able to detect the lowest regulated levels of *L. monocytogenes* and *Salmonella*, those being the absence of both organisms in 25 g of product [34].

The standard culture-based methodology for *S. enterica* and *Listeria* spp. takes three and four days respectively, with confirmatory testing taking further time. In the case of *Listeria* spp., this period can be significantly longer for a confirmed negative. Considering the stringency of EU legislation regarding both *L. monocytogenes* and *S. enterica*, and the small sample volume able to be tested by current molecular methods, it is impossible to remove all culture-based stages (and therefore all of the time) required for this process. Thus minimisation of this culture stage is critical to reducing the total time taken to test of any methodology [40]. Consequently, the selection of the appropriate media for enrichment in studies such as these is key, especially where co-enrichment of organisms differing significantly in their optimal growth conditions is required. Our selection of a modified SEL medium [41] over other universal pre-enrichment broth (UPB) [42] for co-enrichment in our milk-based experiments was based on the necessary inclusion of *E. coli* in our inoculum and the time required for culture. Although Nam *et al.* [43] showed that both *Listeria* and *Salmonella* spp. could grow in the presence of *E. coli* O157 in UPB, Kim & Bhunia [44] showed improved growth over UPB of both target organisms after 16 h co-culture in the presence of *E. coli* in SEL. Our modification of SEL i.e. removal of phosphomycin, allowed for the best culture-based detection of both target organisms, and the use of phosphomycin to inhibit the growth of non-target organisms we found to be counter-productive. A non-target organism, *E. coli*, was used as standard in our test matrix inoculum, so as to approximate the presence of other competing bacteria within our foodstuff and more importantly in our enrichment process. Equally, the use of varying ratios of inoculated bacteria again was performed to more realistically simulate differing levels of contamination. The detection of one or other target species could reasonably be expected in cases of higher contaminating numbers of either target organism, and the media used is designed to reduce the proliferation of non-target organisms in culture.

We arrived at a methodology that best achieved a complete level of detection within a 20 h ‘sample to result’ timeframe. This matches current off-the-shelf individual testing methodologies, but allows for a reduction in the total number of tests required and therefore costs. It is likely that further improvements to this methodology may be possible with the adoption of dye set B and recalibration of the PCR in response to this change. Additionally, removal of some lesser performing amplicons or TRFs would likely further improve results. Regardless, our proposed method remains an approach which is relatively cheap, rapid, and allows for a reduction in tests required due to its co-enrichment basis and multiplex nature. This or similar approaches may be also effective elsewhere in the food production environment or in other areas of environmental health.

Although approaches such as microarrays allow for a higher level of multiplex analysis [44], [45], effective and cheap approaches which can be directly and routinely used are lacking. Quantitative i.e. real-time PCR offers an alternative approach to our methodology, with many methods published [6], [46], [47] and several existing as off-the-shelf products. However, the limitation of a different dye for each pathogen to be detected, and the required significant yet consistent differences of significant length between target species sequences means that closely related species cannot in many cases be differentiated, and numbers of targets are extremely limited by design. The recent study of Wierner *et al.* [47] shows the potential for the successful simultaneous detection of multiple genera using four separate genes, one for each genera, but no allowance can be made for more than one marker for any genus, and with the advent of new six-dye machines, five targets using this process will remain the limit for some time. In our proposed method we have eight markers, three for *S. enterica* and five for *L. monocytogenes*, and twelve potential markers when all seven target species are considered, and the limits in terms of target differentiation are much wider. The ability to target more genes/sequences without loss of sensitivity means that faster and more extensive screening can be performed at reduced cost. Thus we present this approach as a reliable, rapid and cost effective tool for both food and environmental pathogen detection and identification. This opens possibilities for more comprehensive screening and testing programmes and so safer food manufacturing processes and better protection of human health.

## Materials and Methods

### Bioinformatics Analysis and Primer Design

Sequences were selected for use in the differentiation of species from the NCBI database (National Centre for Biotechnology Information, U.S. National Library of Medicine, Bethesda MD, US. www.ncbi.nlm.nih.gov) on the basis of three criteria: i) Good representation of sequence type of a suitable length (>500 bp) for each target species to be differentiated; ii) Presence of regions suitably similar for minimisation of number of oligonucletide primers required for fully inclusive amplification of target species; iii) Presence of endonuclease restriction sites between oligonucleotide primer sites sufficient to discriminate different species amplified. Descriptions of all primers designed for species discrimination or for internal control (IAC) amplification are listed in Table 1 along with the fluorescent dyes used in each set of experiments. All *in vitro* experimental approaches were primarily designed using online sequence databases *in silico*. Genetic sequences of genera/species to be amplified and differentiated were selected from NCBI along with those of closely related genera/species for analysis of inclusivity and exclusivity.

Alignments of selected available representative sequences were performed using the KODON software (Applied Maths, Kortrijk, Belgium) and regions were identified which could be used in the design of oligonucleotide primers that would be expected to amplify all target sequences. Sequence analysis was performed on these alignments in terms of restriction site presence/absence by eye so as to identify the correct combination of restriction sites and suitable primer site availability. This approach ensured maximal inclusivity and exclusivity of all target sequences (where applicable) in the amplification stage along with maximal differentiation by restriction digestion following amplification. Minimisation of the numbers of both oligonucleotide primers and restriction enzymes used as well as minimisation of degeneracy were also important factors in primer site and restriction enzyme selection.

### DNA Extraction

For DNA extraction from pure cultures, all bacterial strains (Table 3) were grown on appropriate solid media and under standard incubation conditions to allow single colony selection for liquid culture and DNA extraction for all species. DNA was extracted from 1 ml of resulting overnight cultures using the Chelex resin method (Bio-Rad Laboratories, Inc., Hercules, CA, US) according to the manufacturer’s instructions. Alternatively, where stated, 1 ml of culture which was centrifuged at 13,000 g for 3 min with the resulting pellet resuspended in 200 µl of PBS and the DNA extracted using the Wizard SV Genomic DNA purification system following the manufacturer’s instructions (Promega, Southampton, UK).

**Table 3:** Strains used *in vitro* for M-TRFLP testing.

Species (n)	Strain ID
*Aeromonas hydrophila*	MIAH1
*Citrobacter braakii*	MICB1
*Citrobacter freundii*	NCTC9750
*Citrobacter koserei*	MICK1
*Clostridium perfringens* (2)	MICP1; MICP2
*Enterobacter cloacae*	NCIMB8556
*Escherichia coli* (10)	ATCC12210*; MIEC2; MIEC3; MIEC4; MIEC48; MIEC50; MIEC52; MIEC54*; MIEC60*; MIEC61*; MIEC66*
*Klebsiella oxytoca*	MIKO1
*Listeria grayi* (2)	CMCC3362; NCTC10815
*Listeria innocua* (8)	CMCC3369; CMCC3370; NCTC11288; MILI4546*; NCTC2159; NCTC21609; ATCC51742^ = ^; MILI1GS
*Listeria ivanovii* (2)	CMCC3365; NCTC11846
*Listeria monocytogenes* (33)	CMCC2993*; CMCC3359; NCTC4883; NCTC4885; NCTC5105; NCTC7973; NCTC7974; NCTC9863; NCTC10357; NCTC10527; NCTC10528; NCTC10887; NCTC10890; NCTC11994*; MILM1*; MILM2; MILM3*; MILM4*; MILM5; MILM5241A; MILMF08120026; MILMQC1680; MILM32; MILMGCC321; MILMLX2; MILM966(6); MISS53; MILMMS3(5); MILM1/236; MILMT46(4); MILMLX9; MILMLX11; MILMSCOTTA
*Listeria murrayi* (2)	CMCC3361; NCTC10812
*Listeria seeligeri* (4)	CMCC3363; NCTC11856; NCTC11289; MILSSS80;
*Listeria welshimeri* (2)	CMCC3366; NCTC11857
*Proteus mirabilis* (6)	MIPM1; MIPM2; MIPM3; MIPM4; MIPM5; MIPM6
*Salmonella enterica* (12)	MISE1*; MISE6*; MISE953807; MISE958149; MISE956110*; MISE807439*; MISE007*; MISE13284; CMCC3750; CMCC3759; CMCC1143; NCTC5791
*Staphylococcus aureus*	CMCC2360
*Vibrio parahaemolyticus* (3)	SPRC10290; MIVPDI-B9; TX2103
*Vibrio vulnificus*	MIVV1
*Yersinia enterocolitica* (7)	NCIMB2124; NCIMB349; NCIMB393; NCIMB441; NCIMB650; NCIMB786; NCIMB844
*Yersinia frederiksenii*	MIYF1
*Yersinia ruckeri* (2)	FDL39/81; MIYR2

* Used in milk inoculation experiments. ATCC: American Type Culture Collection, USA.; CMCC: Colworth Microbiology Culture Collection, Unilever, UK.; NCIMB and FDL: NCIMB Ltd, Aberdeen, UK.; NCTC: National Collection of Type Cultures, U.K.; SPRC and TX: Food and Drug Administration, U.S.A.; MI: This study.

### Bacterial Strains and Culture for Milk Inoculation Experiments

All bacterial strains used are shown in Table 3. Fifteen of these strains were used for milk inoculation and enrichment testing, five each of *E. coli* (EC), *L. monocytogenes* (LM), and *S. enterica* (SE), as highlighted in Table 3. For these experiments, all strains were grown individually overnight at 37°C, with SE and EC strains grown in CM0001 nutrient broth and LM grown in CM129 tryptone soya broth (both Oxoid Ltd., Basingstoke, UK). An EC strain mix were prepared by combining 1 ml of overnight culture of each of the five EC strains and this was repeated for LM and SE strains. Decimal dilutions of each of the three strain mixes were prepared down to a 10^−7^ dilution and 100 µl of both 10^−6^ and 10^−7^ dilutions were plated on non-selective solid media (SE and EC using plain agar (in house) and LM using tryptone soya broth with 0.6% L21 yeast extract and 1.5% LP0013 agar added (Oxoid Ltd., Basingstoke, UK)) in triplicate to estimate cell numbers, with 30 to 50 cfu/ml targeted for the lower dilution. An aliquot (225 µl) of the appropriate strain mix and dilution was added to 25 ml of pasteurised, 2% fat milk at the desired dilution in triplicate to give ratios of SE:EC:LM added (respectively) as 1∶1:1 (at both 10^−6^ and 10^−7^ dilutions), 10∶1:1, 1∶10:1, 1∶1:10, 10∶10:1, 10∶1:10 and 1∶10:10, as well as appropriate no milk and no inoculum controls, again in triplicate. All inoculated milk samples were thoroughly mixed by repeated inversion, and then 10 ml of each was added to 90 ml of modified SEL broth [41], the difference being a lack of phosphomycin in our broth. This was incubated with shaking for 16 hrs at 30°C before sub-samples were taken for culture and for DNA extraction as below. All experiments were repeated independently for verification where appropriate.

### PCR Amplification and Analysis

Genomic template (20 ng) DNA was amplified, unless otherwise stated, using 0.4 mM dNTPs, 0.5 units of Taq (Bioline Reagents Ltd., London, UK), 2 mM MgCl_2_, 1×NH_4_ buffer as supplied (Bioline Reagents Ltd., London, UK) and 0.4 µg/µl BSA in each reaction. The control DNA sequence from the ABI PRISM® dGTP BigDye™ Terminator v3.0 Ready Reaction Cycle Sequencing Kit (Applied biosystems, Foster City, California, US) this being a fragment of the pGEM-3Zf(+) plasmid (Promega Corporation, Madison, Wisconsin, US) was also included in the amplification mix at a final concentration of 13 pg/ml to act as an internal amplification control (IAC). For this IAC, primers were designed to amplify a region 277 bp (Table 1). Primers were used as a 15-primer multiplex PCR, with final concentrations as follows: PrsListFwd and PrsListRevA at 600 nM; PrsListRevB, RecListFwd3 and RecListRev at 333 nM; FusSalFwd, FusSalRev, RpoSalmFwd2, RpoSalmRevB, PrsListFwd2, PrsListRevC, HlyFwd2 and HlyRev2 at 267 nM; and IacFwd and IacRev2 at 67 nM. Primers were 5¢ labelled using fluorescent dyes as described in Table 1, completely using either dye set A or dye set B. Amplification was performed as follows: 95°C for 5 min followed by 30 cycles of 94°C for 30 sec, 55°C for 30 sec and 72°C for 60 sec, and the reaction completed with a final elongation step of 72°C for 10 min. When combinations of three template species per reaction were used, one was always an SE template. When combinations of two templates only were used, one was always one of either the SE or the LM. In this way all possible combinations of two- and three-way mixes gave 15 different three-way and 11 different two-way combinations for the seven target species DNA. All two-way mixes were combined in a 10∶1 ratio, with the SE (or LM in the absence of SE) being the higher amount. Various additional ratios of the three-way mixes were used leading to a total of 42 three-way mixes being analysed. In all cases of template mix ratios being used, ‘1’ represents 2 ng of template DNA and ‘10’ 20 ng template DNA per 30 ul reaction.

### PCR Product Purification, Digestion and M-TRFLP Analysis

When performed, amplification products were purified using the UltraClean PCR clean-up kit (MO BIO Laboratories, Inc., Carlsbad, California, US) according to the manufacturer’s instructions, except that DNA was eluted in 30 µl rather than 100 µl elution buffer. Otherwise samples were digested directly from PCR. Sample concentrations were estimated by gel electrophoresis and either ca. 50 ng or ca. 200 ng were digested dependent on whether the product was from a single strain or from multiple strains respectively. All samples were digested using *Hha*I (Promega, Southampton, United Kingdom) as previously described [29]. When dye set A was used, 2 ul of the digest was mixed with 0.3 µl of TAMRA-labelled GS500 internal size standard and 12 ul of formamide (all reagents obtained from Applied Biosystems, Warrington, United Kingdom). When dye set B was used, the internal standard was changed to LIZ-labelled GS500 (−250) (Table 1). Prior to fragment analysis, samples were denatured at 95°C for 5 min and then chilled on ice for 5 min. Fragment size analysis was carried out with an ABI PRISM 3130*xl* Genetic Analyzer (Applied Biosystems, Warrington, United Kingdom).

### Data Analysis

M-TRFLP profiles were produced using the GeneMapper software (version 3.7; ABI, United Kingdom). Terminal restriction fragments were quantified using the advanced mode and second-order algorithm. Only peaks at positions between 50 and 500 bp were within the linear range of the internal size standard used and were therefore considered. All peaks with heights that were less than 0.5% of the total peak height were ignored in the post-PCR purified samples, but in those samples that were not purified, a cut-off of 60 bp was applied due to primer-dimer presence impacting total peak measurement. If there was no detection of the internal amplification control (IAC) in any profile, even if other TRFs were detected, this indicated a lack of efficient amplification and the sample was excluded.
